# Retracted: The Study of Fetal Rat Model of Intra-amniotic Isoproterenol Injection induced Heart Dysfunction and Phenotypic Switch of Contractile Proteins

**DOI:** 10.1155/2022/9867191

**Published:** 2022-03-21

**Authors:** BioMed Research International

*BioMed Research International* has retracted the article titled “The Study of Fetal Rat Model of Intra-amniotic Isoproterenol Injection induced Heart Dysfunction and Phenotypic Switch of Contractile Proteins” [1] as it was found to contain duplicated figures.

As raised on PubPeer [2], in Figure 3, the Iso1 panel overlaps with the Sham2 panel and in Figure 4 the Sham1 panel overlaps with the Control panel. The authors say this was due to inadvertently selecting the wrong files at revision and they provided the original images on the Open Science Framework [3]; the images in Figures 2 and 3 were changed at the second revision, but the images in Figure 4 are the same as initially submitted.

The editorial board recommend retraction. After the authors were informed of the decision, they agreed with retraction due to the errors identified.
